# Effect of Metformin and Sitagliptin on Doxorubicin-Induced Cardiotoxicity in Rats: Impact of Oxidative Stress, Inflammation, and Apoptosis

**DOI:** 10.1155/2015/424813

**Published:** 2015-12-31

**Authors:** Mina Thabet Kelleni, Entesar Farghaly Amin, Aly Mohamed Abdelrahman

**Affiliations:** Department of Pharmacology, Faculty of Medicine, Minia University, Minia, Egypt

## Abstract

Doxorubicin (DOX) is a widely used antineoplastic drug whose efficacy is limited by its cardiotoxicity. The aim of this study was to investigate the possible protective role of the antidiabetic drugs metformin (250 mg/kg dissolved in DW p.o. for seven days) and sitagliptin (10 mg/kg dissolved in DW p.o. for seven days) in a model of DOX-induced (single dose 15 mg/kg i.p. at the fifth day) cardiotoxicity in rats. Results of our study revealed that pretreatment with metformin or sitagliptin produced significant (*P* < 0.05) cardiac protection manifested by a significant decrease in serum levels of LDH and CK-MB enzymes and cardiac MDA and total nitrites and nitrates levels, a significant increase in cardiac SOD activity, and remarkable improvement in the histopathological features as well as a significant reduction in the immunohistochemical expression of COX-2, iNOS, and caspase-3 enzymes as compared to DOX group. These results may suggest using metformin and/or sitagliptin as preferable drugs for diabetic patients suffering from cancer and receiving DOX in their chemotherapy regimen.

## 1. Introduction

Doxorubicin (DOX) is a common antineoplastic anthracycline antibiotic and is used for treatment of many types of cancer [1]. However, the risk of cardiac, renal, pulmonary, testicular, and hematological toxicities largely limits its effective and widespread use in clinical oncology [2]. The heart is more susceptible to DOX-induced lipid peroxidation and toxicity because of its high energy requirement and high mitochondrial density. Further, the heart also lacks the antioxidant enzymes needed to detoxify superoxide anions and hydrogen peroxide; thus, the generated free radicals accumulate and cause severe lipid peroxidation, leading to extensive destruction of the cardiac cellular mitochondrial membranes, endoplasmic reticulum, and nucleic acid [1, 3].

The pathophysiological background of DOX cardiotoxic effect is multifactorial and not completely elucidated [3, 4]. One of the likely mechanisms is the oxidative injury evoked by formation of DOX iron complex which eventually causes cardiac dysfunction through oxidizing lipids, proteins, and DNA. This damage produced by DOX is dose-related and may lead to cardiomyopathy [5, 6].

Metformin is a well-known biguanide approved for treatment of type 2 diabetes mellitus (T2DM). Metformin was also shown to possess antioxidant and anti-inflammatory properties [7]. Similarly, sitagliptin is an antidiabetic drug acting via inhibiting the enzyme dipeptidyl peptidase-4 and increasing the release of insulin inside the body [8]. Moreover, sitagliptin increases beta-cell proliferation and glucose-stimulated insulin secretion in humans [9]. Further, sitagliptin was shown to possess antioxidant and anti-inflammatory properties [10, 11]. Our study was a trial to assess the possible protective effects of the antidiabetic drugs metformin and sitagliptin in a rat model of DOX-induced cardiotoxicity.

## 2. Materials and Methods

### 2.1. Animals, Experimental Design, and Drugs

Male Wistar rats were randomly distributed into six (6) groups of six to nine rats. Further details of groupings and treatments are as follows:

 Control: received distilled water (DW) p.o.
 Metformin: received metformin at a dose of 250 mg/kg/day p.o. for seven days.
 Sitagliptin: received sitagliptin in a daily dose of 10 mg/kg/day p.o. for seven days.
 Doxorubicin: received DOX in a single dose of 15 mg/kg i.p. at the fifth day.
 Metformin/DOX: received both metformin and DOX as described.
 Sitagliptin/DOX: received both sitagliptin and DOX as described.

All animals had free access to food and water ad libitum and lighting was maintained at a 12 h cycle. All animal care and experimental procedures were in accordance with the protocols of the Research Advisory Ethical Committee of Faculty of Medicine, Minia University, Egypt.

The drugs used were metformin powder (CID, Egypt), sitagliptin powder (Wuhan Golden Wing Industry & Trade Co., Ltd., China), and doxorubicin (Oncodox-50 vials; Cipla Ltd., India).

### 2.2. Collection of Blood and Heart Specimens

At the end of treatments, animals were weighed and sacrificed. The blood samples were collected and serum was separated by centrifugation to be stored at −80°C and thawed just before the biochemical analysis. The heart tissues were excised and weighed and a part taken of each heart tissue was fixed in 10% formalin for histopathological examination and immunohistochemical enzymatic assay while the other part was kept in −80°C and thawed just before homogenization in phosphate buffered saline for the biochemical assay.

### 2.3. Analytical Methods

#### 2.3.1. LDH and CK-MB

Serum levels of lactate dehydrogenase (LDH) and creatine kinase-MB isoenzyme (CK-MB) enzymes were determined according to the guidelines of some locally available commercial kinetic kits using the principles previously described by Buhl and Jackson and Wu and Bowers Jr., respectively [12, 13].

#### 2.3.2. MDA

Cardiac malondialdehyde (MDA) level was detected biochemically. Trichloroacetic acid was added to the sample for protein precipitation and then thiobarbituric acid was added. The mixture was heated for 10 min in a boiling water bath. One molecule of MDA in the homogenized heart samples reacted with two molecules of thiobarbituric acid and the resulting chromogen was centrifuged. The intensity of the color developed in the supernatant was measured spectrophotometrically at 535 nm [14, 15].

#### 2.3.3. NO_*x*_

Cardiac total nitrites and nitrates (NO_*x*_) level was detected biochemically. Nitric oxide (NO) is rapidly oxidized to nitrite and/or nitrate by oxygen and the stable oxidation end products of NO, nitrite, and nitrate were used as an index of NO production. The method used to determine NO_*x*_ in homogenized heart samples depends on reduction of nitrate by copper-cadmium granules, followed by color development with Griess reagent (sulfanilamide and *N*-naphthylethylenediamine) in acidic medium to be measured spectrophotometrically at 540 nm [16].

#### 2.3.4. SOD

Cardiac superoxide dismutase (SOD) activity was detected biochemically. The method used to determine SOD activity in homogenized heart samples is based on the fact that the autoxidation of pyrogallol is inhibited by SOD. One unit of SOD is generally defined as the amount of enzyme that inhibits the autoxidation of pyrogallol by 50%. The activity of SOD was monitored spectrophotometrically at 420 nm [17].

#### 2.3.5. Histopathology and Immunohistochemistry

Histopathological examination and immunohistochemical measurement of cyclooxygenase-2 (COX-2), inducible nitric oxide synthase (iNOS), and caspase-3 enzymatic expression were performed with the help of a pathologist. Histopathological changes were graded as 0 (absent), 1 (mild), 2 (moderate), and 3 (severe) [18]. Semiquantitative scoring of COX-2 and iNOS enzymes was done by determining immunoreactivity under light microscope magnification ×200. The cell dyeing conditions were evaluated and classed as follows: the blank dyeing is zero; the focal dyeing is one; the mild diffuse dyeing is two; the moderate diffuse dyeing is three; the strong diffuse dyeing is four [19]. Similarly, caspase-3 semiquantitative scoring was done by determining immunoreactivity under light microscope magnification ×100. Staining intensity was scored as 0 (negative), 1 (weak), 2 (moderate), and 3 (strong). Staining extent was scored as 0 (0%), 1 (1–25%), 2 (26–50%), 3 (51–75%), and 4 (76–100%) according to the percentage of cells staining positive for active caspase-3 [20].

### 2.4. Statistical Evaluation

Results were shown as mean ± SEM of six observations. One-way analysis of variance (ANOVA) with Tukey's multiple comparison test was used to find statistical significance (*P* < 0.05).

## 3. Results

**Figure 1:**
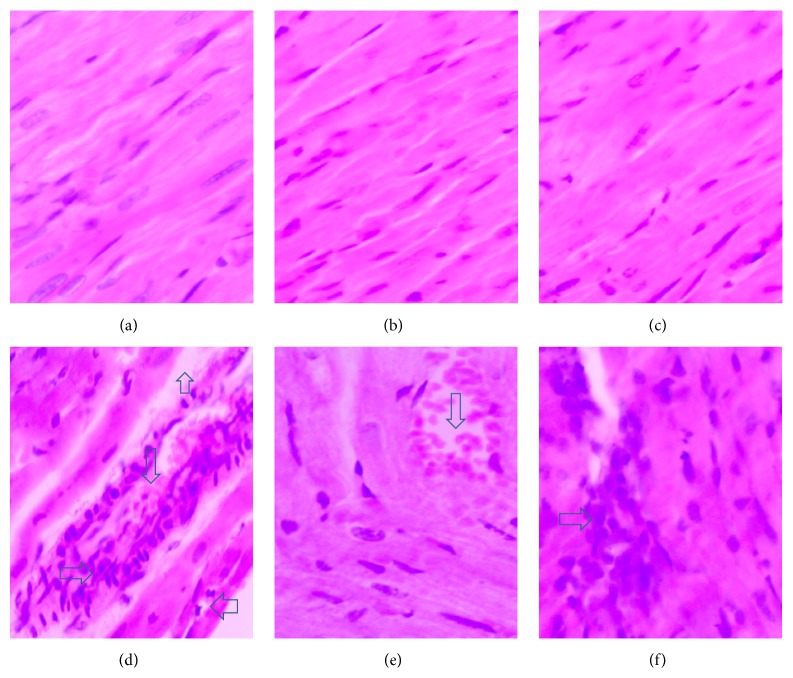
Photomicrography of histopathological changes in the studied different animal groups. (a), (b), and (c) represent normal cardiac tissue sections from the control, metformin, and sitagliptin groups, respectively. (d) shows extensive myocardial degeneration (up arrow), perinuclear vacuolization (left arrow), inflammatory cells (right arrow), and interstitial hemorrhage (down arrow) found in the DOX group. (e) and (f) show significant improvement in the pathological features in the metformin/DOX and sitagliptin/DOX groups, respectively, as compared to (d). Sections were stained with H&E and magnification used was 200x.

**Figure 2:**
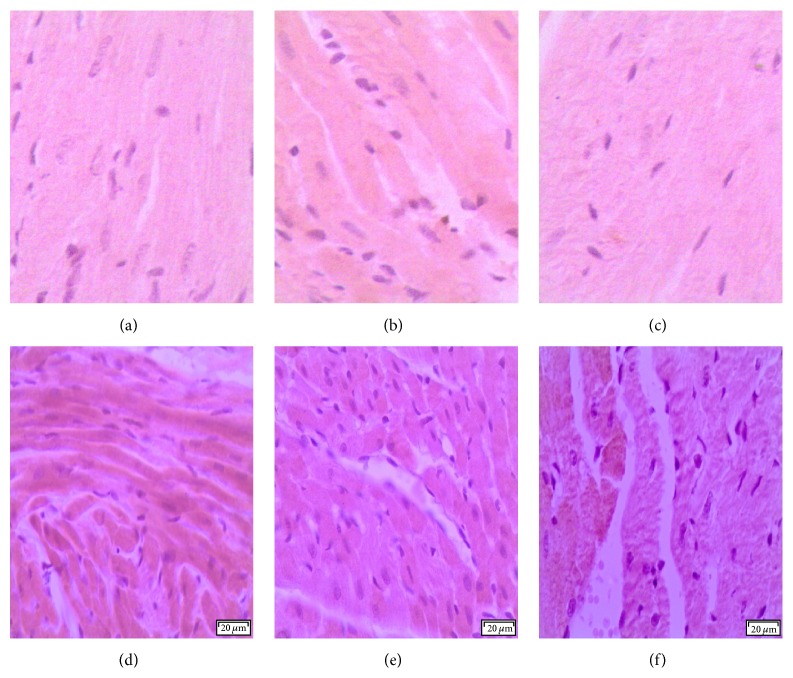
Photomicrography of COX-2 immunostaining in the studied different animal groups. (a), (b), and (c) represent minimal expression sections from the control, metformin, and sitagliptin groups, respectively. (d) represents high COX-2 expression found in the DOX group. (e) and (f) represent significantly lower COX-2 expression found in the metformin/DOX and sitagliptin/DOX groups, respectively, as compared to (d). DAB was used as a chromogen and haematoxylin as nuclear and counter stain. Magnification used is 200x.

**Figure 3:**
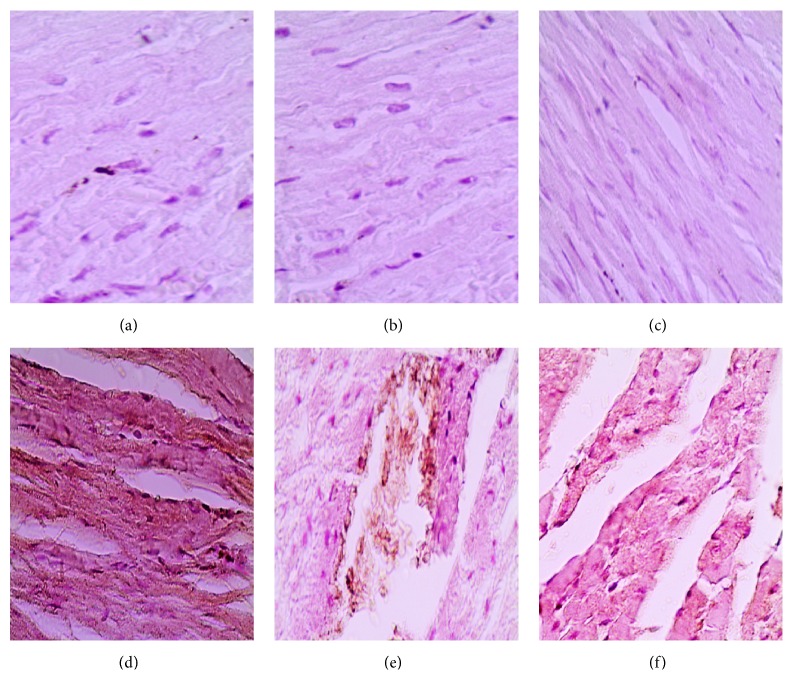
Photomicrography of iNOS immunostaining in the studied different animal groups in the third set. (a), (b), and (c) represent minimal expression sections from the control, metformin, and sitagliptin groups, respectively. (d) represents high iNOS expression found in the DOX group. (e) and (f) represent significantly lower iNOS expression found in the metformin/DOX and sitagliptin/DOX groups, respectively, as compared to (d). DAB was used as a chromogen and haematoxylin as nuclear and counter stain. Magnification used is 200x.

**Figure 4:**
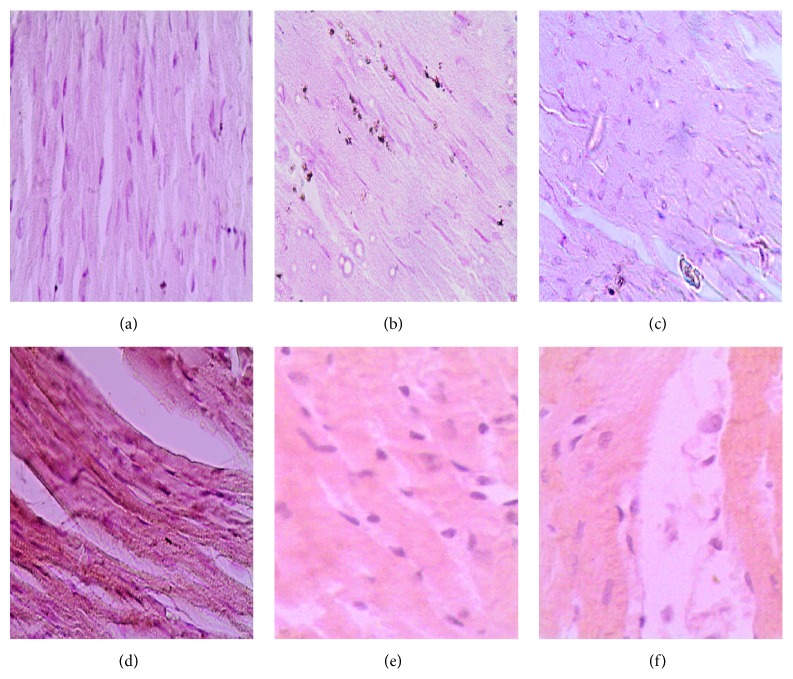
Photomicrography of caspase-3 immunostaining in the studied different animal groups. (a), (b), and (c) represent minimal expression sections from the control, metformin, and sitagliptin groups, respectively. (d) represents high caspase-3 expression found in the DOX group. (e) and (f) represent significantly lower caspase-3 expression found in the metformin/DOX and sitagliptin/DOX groups, respectively, as compared to (d). DAB was used as a chromogen and haematoxylin as nuclear and counter stain. Magnification used is 200x.

DOX administration produced significant elevation in serum levels of LDH and CK-MB enzymes and cardiac levels of MDA and NO_*x*_ and produced a significant reduction in cardiac SOD activity compared with the control group (Table 1). Pretreatment with either metformin or sitagliptin has significantly (*P* < 0.05) attenuated DOX-induced cardiotoxicity manifested by a significant reduction (*P* < 0.05) in serum levels of LDH and CK-MB as well as cardiac levels of MDA and NO_*x*_. Further, cardiac SOD activity was significantly (*P* < 0.05) restored in metformin/DOX and sitagliptin/DOX groups compared with DOX group. Interestingly, no significant change was noticed for DOX, metformin, or sitagliptin regarding their effect on the final body weight or relative heart weight (heart versus body weight ratio, mg/g). The data represented in Table 2 and Figure 1 reveal extensive areas of degeneration, perinuclear vacuolization, inflammation, and interstitial hemorrhage in DOX group (Figure 1(d)). These changes were significantly attenuated in metformin/DOX group (Figure 1(e)) as well as in the sitagliptin/DOX group (Figure 1(f)). Immunohistochemical enzymatic expression in the form of brown spots was significantly (*P* < 0.05) increased in the DOX group compared with the control group. Interestingly, there was a significant (*P* < 0.05) decrease in COX-2, iNOS, and caspase-3 expressions in metformin/DOX group as well as sitagliptin/DOX group as compared to DOX group (Table 3, Figures 2, 3, and 4).

## 4. Discussion

Anthracycline antibiotics, including DOX, may cause myocardial dysfunction in up to 25% of patients and life-threatening heart failure in 1–4% of patients. However, they are widely used in antineoplastic protocols because remission rates with these agents are superior to those of many other chemotherapeutic agents. DOX-induced cardiotoxicity was shown to be dose-related. Doses exceeding 500 mg/kg are commonly associated with left ventricular dysfunction and development of clinically overt congestive heart failure [21, 22]. Increased apoptosis, generation of reactive oxygen species, and disturbances in mitochondrial calcium homoeostasis as well as preferential accumulation of iron inside the mitochondria following DOX treatment were suggested to explain its cardiotoxicity [6, 23].

The main aim of this study was to investigate the possible protective potentials of the antidiabetic drugs metformin and sitagliptin against the development of DOX-induced cardiotoxicity in rats.

In the present work, DOX cardiotoxicity was induced by its administration in a single i.p. dose of 15 mg/kg [24]. Cardiotoxicity was manifested by significant elevation of serum levels of CK-MB and LDH and confirmed by histopathological and immunohistochemical examinations.

Data represented in our study showed that DOX-induced cardiotoxicity in rats was associated with significant elevation of serum levels of LDH and CK-MB enzymes compared with the control group. Elevation of LDH and CK-MB levels represents their leakage from the damaged membranes of cardiomyocytes into the circulation and was previously shown to be an indicator for cardiotoxicity [25, 26]. Our results revealed that pretreatment with metformin or sitagliptin significantly attenuated the rise of LDH and CK-MB. In accordance with our results, Chang et al. demonstrated that sitagliptin pretreatment decreased CK-MB and LDH release in a cardiac ischemia reperfusion (I/R) rat model [27]. Moreover, Ashour and colleagues have shown that DOX cotreatment with metformin eliminated the increase in serum levels of CK-MB and LDH in rats [28]. Our results also show that DOX-induced cardiotoxicity in rats was associated with a significant rise in cardiac MDA and NO_*x*_ levels as well as a significant reduction in cardiac SOD activity. Pretreatment with metformin or sitagliptin significantly decreased the cardiac nitrooxidative stress parameters (MDA and NO_*x*_) and restored the cardiac SOD activity in rats subjected to DOX-induced cardiotoxicity. It is noteworthy that both metformin and sitagliptin were reported to possess antioxidant properties decreasing the accumulation of free radicals; Aleisa and colleagues have shown that pretreatment with metformin protected against the DOX-induced increase in MDA in Swiss albino mice [29]. Similarly, sitagliptin has ameliorated the elevation of MDA and NO_*x*_ levels in a model of endothelium dysfunction induced by atherogenic diet in rabbits [30]. Further, metformin has increased cardiac SOD activity in a model of T2DM genetically modified mice, an effect attributed to its antioxidant free radical scavenging ability [31], and sitagliptin was also shown to increase SOD activity [27].

Interestingly, while metformin has significantly ameliorated the increase of NO_*x*_ as compared to DOX group, it increased NO_*x*_ level as compared to control and sitagliptin groups. It is already well known that NO may represent a double edged sword with pleotropic physiological as well as some serious deleterious effects in case of overproduction [32]. Zhang and colleagues have shown that the improvement in cardiac structure and function following metformin treatment in a rat model of ventricular hypertrophy was associated with enhanced eNOS and increased NO production [33]. Moreover, metformin was also found to decrease the serum NO reduction in a model of vascular endothelial dysfunction induced by low density lipoprotein in rats. The mechanism was suggested to be associated with protection of endothelium-dependent relaxation factor and inhibition of the oxidative stress [34].

In our study, prior administration of metformin or sitagliptin significantly decreased the expression of the proinflammatory COX-2 and iNOS enzymes as well as the proapoptotic executioner caspase-3 enzyme compared with DOX group. In accordance with our results, metformin treatment has significantly prevented the increase of COX-2 and iNOS expression in a rat model of endotoxin-induced uveitis in rats [35] as well as in a rat model of ovarian hyperstimulation syndrome [36]. Furthermore, metformin prevented the activation of caspase-3 when administered 24 hours prior to DOX administration in a cell line of cardiomyocytes [37]. Similarly, sitagliptin has downregulated COX-2 expression in arteries of spontaneously hypertensive rats [38]. Further, sitagliptin decreased the mRNA expression of iNOS in a model of ovalbumin-induced murine model of allergic airway disease [39] and it also reduced the key downstream executioner caspase-3 expression in a recent study showing the ability of sitagliptin to attenuate transient cerebral I/R injury in diabetic rats [40].

## 5. Conclusion

In summary, the present findings reveal that pretreatment with either metformin or sitagliptin has significantly attenuated DOX-induced cardiotoxicity in rats due to antioxidant, anti-inflammatory, and antiapoptotic properties. Metformin and/or sitagliptin may be suggested to be preferable drug(s) for diabetic patients suffering from cancer and receiving doxorubicin as a component of their chemotherapy regimen. Further studies to elucidate more of their mechanisms and potentials are advised.

## Figures and Tables

**Table 1:** Effect of metformin or sitagliptin on DOX-induced cardiotoxicity in rats.

Parameter	Control	Metformin	Sitagliptin	DOX	Metformin/DOX	Sitagliptin/DOX
Heart/body weight ratio (mg/g)	2.91 ± 0.04	3.08 ± 0.09	2.85 ± 0.1	2.96 ± 0.01	2.86 ± 0.05	3.1 ± 0.07
LDH (IU/L)	469.12 ± 4.91	427.82 ± 4.26	448.48 ± 5.62	2199.3 ± 33.25^a^	666.7 ± 10.88^ab^	1171.9 ± 19.47^ab^
CK-MB (IU/L)	537.98 ± 3.9	506.2 ± 5.05	518.72 ± 5.58	2632.78 ± 53.29^a^	715.1 ± 31.84^ab^	1021.97 ± 27.3^ab^
MDA (nmol/g tissue)	28.7 ± 0.54	27.68 ± 0.41	28.63 ± 0.43	70.77 ± 1.22^a^	50.9 ± 0.87^ab^	54.85 ± 1.28^ab^
NO_*x*_ (nmol/g tissue)	127.47 ± 0.77	157.48 ± 1.61^a^	126.71 ± 0.84	304.36 ± 6.45^a^	228.87 ± 7.72^ab^	213.26 ± 5.95^ab^
SOD (U/mg tissue)	0.88 ± 0.04	0.89 ± 0.04	0.89 ± 0.04	0.39 ± 0.04^a^	0.74 ± 0.04^b^	0.59 ± 0.03^ab^

LDH: lactate dehydrogenase; CK-MB: creatine kinase-MB isoenzyme; MDA: malondialdehyde; NO_*x*_: total nitrites and nitrates; SOD: superoxide dismutase. Results are expressed as mean ± SEM of six observations.

^a^Significantly different from control group (*P* < 0.05). ^b^Significantly different from DOX group (*P* < 0.05).

**Table 2:** Effect of metformin or sitagliptin on the histopathology in DOX-induced cardiotoxicity in rats.

Parameter	Control	Metformin	Sitagliptin	DOX	Metformin/DOX	Sitagliptin/DOX
Myocardial degeneration	0 ± 0	0 ± 0	0 ± 0	2.00 ± 0.27^a^	0.25 ± 0.04^b^	0.50 ± 0.03^b^
Interstitial inflammation	0 ± 0	0 ± 0	0 ± 0	2.75 ± 0.32^a^	0.50 ± 0.02^b^	1.00 ± 0.07^ab^
Interstitial hemorrhage	0 ± 0	0 ± 0	0 ± 0	2.25 ± 0.21^a^	0.75 ± 0.03^ab^	0.50 ± 0.02^b^

Data were expressed as mean ± SEM of six observations.

^a^Significantly different from control group (*P* < 0.05).

^b^Significantly different from doxorubicin group (*P* < 0.05).

**Table 3:** Effect of metformin or sitagliptin on the immunohistochemical enzymatic expression of COX-2, iNOS, and caspase-3 in DOX-induced cardiotoxicity in rats.

Parameter	Control	Metformin	Sitagliptin	DOX	Metformin/DOX	Sitagliptin/DOX
COX-2	1.25 ± 0.42	1.50 ± 0.54	1.25 ± 0.23	3.75 ± 0.43^a^	2.00 ± 0.32^ab^	2.50 ± 0.58^ab^
iNOS	1.75 ± 0.28	1.50 ± 0.28	1.50 ± 0.28	3.50 ± 0.35^a^	2.25 ± 0.36^b^	2.75 ± 0.24^ab^
Caspase-3	1.25 ± 0.35	1.00 ± 0.42	1.25 ± 0.36	3.75 ± 0.52^a^	1.75 ± 0.25^b^	2.25 ± 0.37^ab^

Data were expressed as mean ± SEM of six observations.

^a^Significantly different from control group (*P* < 0.05).

^b^Significantly different from doxorubicin group (*P* < 0.05).
